# Attitude and Willingness towards Tissue Donation in Iranian High School Students: Bone Marrow and Blood

**Published:** 2011-11-01

**Authors:** S. Sanavi, R. Afshar, N. Sanavi

**Affiliations:** 1 *Clinical Fellow of Nephrology and Transplantation, Internist, University of Social Welfare & Rehabilitation Sciences Akhavan Center, Tehran, Iran*; 2 *Associate Professor of Nephrology and Transplantation, Shahed University, Mustafa Khomeini Hospital, Tehran, Iran.*; 3 *Bachelor of Science in Statistics, Majlis Research Center, Tehran, Iran.*

**Keywords:** Attitude, Willingness, Donation, Tissue, Bone marrow

## Abstract

Background: Tissue donation has been promising in prolonging the lives of people with life-threatening diseases.

Objective: To assess the attitude and willingness of high school students towards bone marrow (BM) and blood (as tissue) donation for maintaining a safe and adequate pool.

Methods: This cross-sectional study was conducted among the high school girls, aged 15–18 years, who studied in natural sciences, mathematics and humanities. All participants filled a questionnaire consisting of age, religion, education levels and study branch, attitude and willingness towards BM and blood donation according to a Likert scale.

Results: Out of 416 students, with the mean±SD age of 16.3±1.2 years, 31% studied in grade I, 27% in grade II (25% natural sciences, 27% mathematics, and 48% humanities), 26% in grade III (30% natural sciences, 34% mathematics, and 36% humanities), and 16% in pre-university level (college) (32% natural sciences, 42% mathematics, and 6% humanities). The students had highly positive attitudes toward BM and blood donation (mean±SD score of 4.2±0.54). The willingness for BM and blood donation was declared respectively, in 87% and 71% of respondents. Moreover, 16% of students wanted to donate only to their relatives and 84% to all persons in need of therapy. There was no significant correlation between the donation willingness and educational levels and study branch; however, these variables significantly correlated with attitude towards tissue donation (p=0.02, p=0.01, respectively).

Conclusion: Despite positive attitude towards BM and blood donation, willingness for BM donation is lesser than blood which may be attributed to insufficient information about this type of tissue donation. An organized educational program for high school students in all aspects of tissue donation seems necessary.

## INTRODUCTION

Organ and tissue transplantations in recent years play a significant role in prolongation of lives particularly in patients who suffer from chronic debilitating diseases [1, 2]. The major limiting factor in transplantation program is the shortage of donor organs pool. Although the public awareness regarding the solid organ donation has been increasingly upgraded during the recent years, the public information surrounding tissue donation such as bone marrow (BM) is scarce. BM transplantation is feasible by samples drawn from one’s own or relatives and strangers [3]. BM donation is a relatively safe procedure with a few side effects including anesthesia risks and rare infectious and hemorrhagic complications [2]. For instance, the said complications have been reported in 0.5% of cases among 1270 donors in one study [4]. Considering the exigent need for blood transfusion, feasibility of BM donation prior to death, easiness and relative safety of BM donation, and low public awareness about scientific and religious aspects of BM donation, it seems that extensive educational program through the mass media and medical professionals is necessary. It is important that these informative and promotive messages should be completely scientific and not oppose with the public religious beliefs [5]. Such notification must be based on knowledge of the findings of other studies on the status of public attitude and willingness towards organ or tissue donation. This study was therefore conducted to evaluate the public awareness of BM and blood donation among high school students.

## MATERIALS AND METHODS

This study was performed on 416 high school girls aged between 15 and 18 years of Tehran, Iran. Based on a 5-point Likert scale [6-8] and without prior education, the attitude and willingness to donate BM and blood were assessed by an anonymous questionnaire. Attitudes towards tissue donation were assessed by a 5-point Likert scale, with ‘5’ indicating “strongly agree” and ‘1’ depicting “strongly disagree.” The questionnaire consisted of 14 items (7 positive and 7 negative questions towards organ donation). The total score was calculated as the average of all items with the results of negative items inverted (Table 1). Willingness to donate was evaluated by three items: one about donation of one’s own tissues, and two in relation to tissue donation for scientific purposes or helping others. Each item was rated on a 5-point Likert scale from ‘1’ indicating “no willingness” to ‘5’ indicating “absolute willingness.”

Statistical analyses were performed by SPSS v.16. The Spearman’s correlation coefficient and Kruskal-Wallis tests were used for analyses of data. A p value <0.05 was considered statistically significant.

## RESULTS

The study population consisted of 416 girls with the mean±SD age of 16.26±1.06 years and moderate socioeconomic status. The participants came from four grades and three study branches including grade I or general (n=129, 31%); grade II (n=112, 27%); grade III (n=108, 26%); and grade IV or pre-university level or college (n=67, 16%) (Table 2).

Based on a Likert scale, attitude towards tissue donation were highly positive with a mean±SD score of 4.2±0.54. Of the respondents, 87% preferred to donate blood while 71% were willing to donate BM (Table 3). Furthermore, 16% of the participants wanted to donate only to their relatives; 84% agreed to donate to everyone in need. The purpose of tissue donation in 60% of respondents was only helping others; 40% stated both scientific and helping purposes.

There was positive correlation between the attitude towards tissue donation and educational levels and study branches (p=0.02, p=0.01, respectively). However; no significant correlation was found between the willingness for tissue donation and the above variables.

## DISCUSSION

So far, several studies regarding attitude towards organ/tissue donation have been performed. Most research studies showed that although the public generally have a positive attitude towards tissue donation, a small percentage of them actually decide to donate their organs/tissues. Indeed, the relationship between attitudes and ultimate behaviors is not certain [9-13]. In such cases, the population must be informed and encouraged towards organ/tissue donation through the media and medical professionals [5]. Various measures to encourage the general population to donate their tissues/organs have been suggested [14-16]. The most common reason for organ/tissue donation is altruistic motivations. Many researchers believe that proper information surrounding different aspects of transplantation procedure and organ/tissue donation has positive effects on the public decision making [17]. In most cases, a positive incentive to tissue/organ donation arises from altruism or pride feeling due to donation and negative incentive results from fear of mutilation or incomplete medical treatment [18]. 

Currently, according to the national program for BM donation in the US, more than four million subjects have registered to donate BM which indicates increasing public awareness regarding tissue donation [19]. Unfortunately, despite numerous attempts of the charity foundation of special diseases and organ transplantation (CFFSD) in recent years, broadcasting educational programs through the media and publication of many articles in magazines and newspapers in Iran, tissue donation (except for blood) has not yet been greatly popular.

Compared to similar study on medical students of Shahed University in Tehran, this study revealed that high school students without prior education had a high positive attitude (mean±SD scores of 4.2±0.54 *vs*. 4.3±0.46) and willingness toward blood and BM donation (87% and 71% *vs.* 89.3% and 81.6%, respectively). Lesser willingness of high school students toward blood and BM donation may be attributed to the following reasons: 1) Lack of sufficient knowledge about the BM as a tissue, feasibility of donation before death, easiness of taking sample (with insignificant medical complication rates), and the fact that BM donation can cure many fatal diseases; 2) Lack of adequate educational programs allocated to youth in schools; 3) Broadcasting television serials or documentaries that were not scientifically accurate; 4) Lack of adequate information about the Islam orders about tissue donation—for example, 10% of our study population believed that tissue donation had a confliction with their religious beliefs; and 5) lack of a known tissue procurement organization, particularly for BM, in Iran. It should be noted that in contrast to the study on Shahed University medical students, there was a significant relationship between educational levels and attitude towards tissue donation [8]. In addition, the attitude of students differed with their study branches, so that students of humanities had the highest score (4.3±0.84) and students of natural sciences had the lowest (3.8±0.31). These findings may be attributed to more awareness of the students of natural sciences about tissue donation and fear of its medical complications. It seems that they had not enough knowledge about the types and rates of donation complications which indicates the necessity of more programmed education by medical professionals. Considering the long history of knowledge of individuals about blood donation, 87% of respondents were also willing to donate blood that has a significant percentage in comparison with findings of other studies conducted on the general population in Iran (51.1%) [20]. Unwillingness of the remainder may be related to insufficient knowledge about the multiple benefits of blood donation, safety and easiness of donation, and the fact that every person may sometimes need to receive blood to survive, and thus replacement must be provided. Unfortunately, few studies regarding the public attitude and willingness towards tissue donation have been conducted so far; most researches had been mainly focused on medical professionals and staff [7, 20-23]. Another advantage of the present study was that while other studies had qualitative approaches [23-26], this study used a quantitative evaluation of attitude towards tissue donation. Based on what we found, it seems it is necessity to establish an organized educational program explaining the practical, legal, and ethical aspects of tissue donation to general population. 
